# De novo transcriptome sequencing of radish (*Raphanus sativus* L.) fleshy roots: analysis of major genes involved in the anthocyanin synthesis pathway

**DOI:** 10.1186/s12860-019-0228-x

**Published:** 2019-10-23

**Authors:** Jian Gao, Wen-Bo Li, Hong-Fang Liu, Fa-Bo Chen

**Affiliations:** 1 Department of Life Sciences and Technology, Yangtze Normal University, Fuling, 408100 China; 2 Centre for Green Development and Collaborative Innovation in Wuling Mountain Region, Yangtze Normal University, Fuling, China

**Keywords:** Radish *(Raphanus sativus* L.), de novo assembly, RNA-Seq, Anthocyanin biosynthesis related genes (ABRGs), Red pigment

## Abstract

**Background:**

The HongXin radish (*Raphanus sativus* L.), which contains the natural red pigment (red radish pigment), is grown in the Fuling district of Chongqing City. However, the molecular mechanisms underlying anthocyanin synthesis for the formation of natural red pigment in the fleshy roots of HongXin radish are not well studied.

**Results:**

De novo transcriptome of HX-1 radish, as well as that of the advanced inbred lines HX-2 and HX-3 were characterized using next generation sequencing (NGS) technology. In total, approximately 66.22 million paired-end reads comprising 34, 927 unigenes (N50 = 1, 621 bp) were obtained. Based on sequence similarity search with known proteins, total of 30, 127 (about 86.26%) unigenes were identified. Additionally, functional annotation and classification of these unigenes indicated that most of the unigenes were predominantly enriched in the metabolic process-related terms, especially for the biosynthetic pathways of secondary metabolites. Moreover, majority of the anthocyanin biosynthesis-related genes (ABRGs) involved in the regulation of anthocyanin biosynthesis were identified by targeted search for their annotation. Subsequently, the expression of 15 putative ABRGs involved in the anthocyanin synthesis-related pathways were validated using quantitative real-time polymerase chain reaction (qRT-PCR). Of those, *RsPAL2*, *RsCHS-B2, RsDFR1*, *RsDFR2*, *RsFLS*, *RsMT3* and *RsUFGT73B2-like* were identified significantly associated with anthocyanin biosynthesis. Especially for *RsDFR1*, *RsDFR2* and *RsFLS,* of those, *RsDFR1* and *RsDFR2* were highest enriched in the HX-3 and WG-3, but *RsFLS* were down-regulated in HX-3 and WG-3. We proposed that the transcripts of *RsDFR1*, *RsDFR2* and *RsFLS* might be act as key regulators in anthocyanin biosynthesis pathway.

**Conclusions:**

The assembled radish transcript sequences were analysed to identify the key ABRGs involved in the regulation of anthocyanin biosynthesis. Additionally, the expression patterns of candidate ABRGs involved in the anthocyanin biosynthetic pathway were validated by qRT-PCR. We proposed that the transcripts of *RsDFR1*, *RsDFR2* and *RsFLS* might be acted as key regulators in anthocyanin biosynthesis pathway. This study will enhance our understanding of the biosynthesis and metabolism of anthocyanin in radish.

## Background

Anthocyanins are globally recognized as water-soluble pigments that are commonly accumulated in various plant species [1]. Anthocyanins are responsible for imparting the red and purple colours to the plants [2]. Recently, several studies have demonstrated that anthocyanins have vital roles in the plants, including protecting the plant tissues against adverse conditions such as, temperature, irradiation, and photo-oxidative injury [3]. Additionally, anthocyanins are reported to contribute to pollination and thus facilitate seed dispersal in plants [4]. Moreover, anthocyanins can be used as a food additive for protection against various diseases, such as cardiovascular and inflammatory diseases, obesity, and diabetes [5, 6]. Most studies on the anthocyanin biosynthetic pathway have reported that the pathway is largely conserved among the flowering plants [7]. Anthocyanins are synthesized from phenylalanine by several enzymes involved in the phenylpropanoid pathway. The key genes involved in the biosynthesis of anthocyanins encode several enzymes, such as cinnamic 4-hydroxylase (*C4H*), cinnamic 4-coumarate-CoA ligase (*4CL*), and phenylalanine ammonia-lyase (*PAL*) [8, 9]. In the phenylpropanoid pathway, three molecules of malonyl-CoA are sequentially added to one molecule of 4-coumaroyl CoA to yield tetrahydroxychalcone (*THC*), which is catalysed by chalcone synthase (*CHS*), chalcone isomerase (*CHI*), flavonoid 3′-hydroxylase (*F3’H*), and flavanone 3-hydroxylase (*F3H*) [10]. Fukusaki et al. [11] demonstrated that the knockdown of *CHS* gene by RNA interference changes the flower colour from blue to white. Additionally, the *DFR* and *ANS* genes are important regulators of pigmentation and are reported to be involved in the skin pigmentation of mildly coloured pears [12]. The dihydroflavonols 4-reductase (*DFR*) and anthocyanin synthase (*ANS*) genes encode enzymes that can generate various anthocyanidins from dihydroflavonols using NADPH as a cofactor [13]. Next, stable anthocyanidins are formed from the synthesized anthocyanidins encoded by several genes. In grape berry, the gene involved in the anthocyanin biosynthesis is UDP-glucose: flavonoid 3-O-glucosyltransferase (*UFGT*). The absence of *UGFT* gene results in the loss of colour in white grapes [14]. Moreover, several glutathione S-transferases (*GSTs)*, as a large and complex enzyme family (EC 2.5.1.18), played vital roles in plant growth and development and responsive to heavy metal stress, as well as flavonoid metabolism through involved in the sequestration of anthocyanins in many plants, such as grape (*Vitis vinifera*), Arabidopsis (*Arabidopsis thaliana*), and apple (*Malus domestica*) [15–17]. The functions of key anthocyanin biosynthesis-related genes have been extensively characterized. However, the molecular mechanism underlying the regulation of anthocyanin biosynthesis is not fully understood in radish (*Raphanus sativus* L.).

Radish is an annual plant that belongs to the Brassicaceae family and is grown worldwide. In this study, we used the inbred HX-1 line, which is famous for containing natural red pigment (Red Radish pigment), grown in the Fuling district of Chongqing City. The two advanced inbred lines comprised of HX-2 and HX-3 were cultivated and selected from the HX-1 line. The genetic background for these radish genotypes was stably maintained in the homozygous state through self-pollination for many generations. The HX-2, HX-3, and HX-1 genotypes exhibit RW (red skin and white flesh root), RP (red skin and pink flesh root), and RR (red skin and red flesh root) phenotypes, respectively. Recent studies have used the global transcriptome analysis to evaluate the anthocyanin biosynthetic pathway and the expression of anthocyanin biosynthesis-related genes (ABRGs). Several anthocyanin biosynthesis regulatory genes were reported in important fruit crops, such as grape [18], blood orange [19], and blueberry [20]. However, the transcriptome of HX radish inbred lines, such as HX-1, HX-2, and HX-3 and the expression of ABRGs have not been fully investigated.

In this study, the assembled radish transcript sequences were examined to identify the key ABRGs involved in the regulation of anthocyanin biosynthesis. Additionally, the expression patterns of the candidate ABRGs involved in the anthocyanin biosynthetic pathway were validated by quantitative real-time polymerase chain reaction (qRT-PCR). This study will enhance our understanding of the biosynthesis and metabolism of anthocyanin during taproot formation in radish.

## Results

### Assembly and functional annotation of radish “HX-1” fleshy root transcriptome

**Fig. 1:**
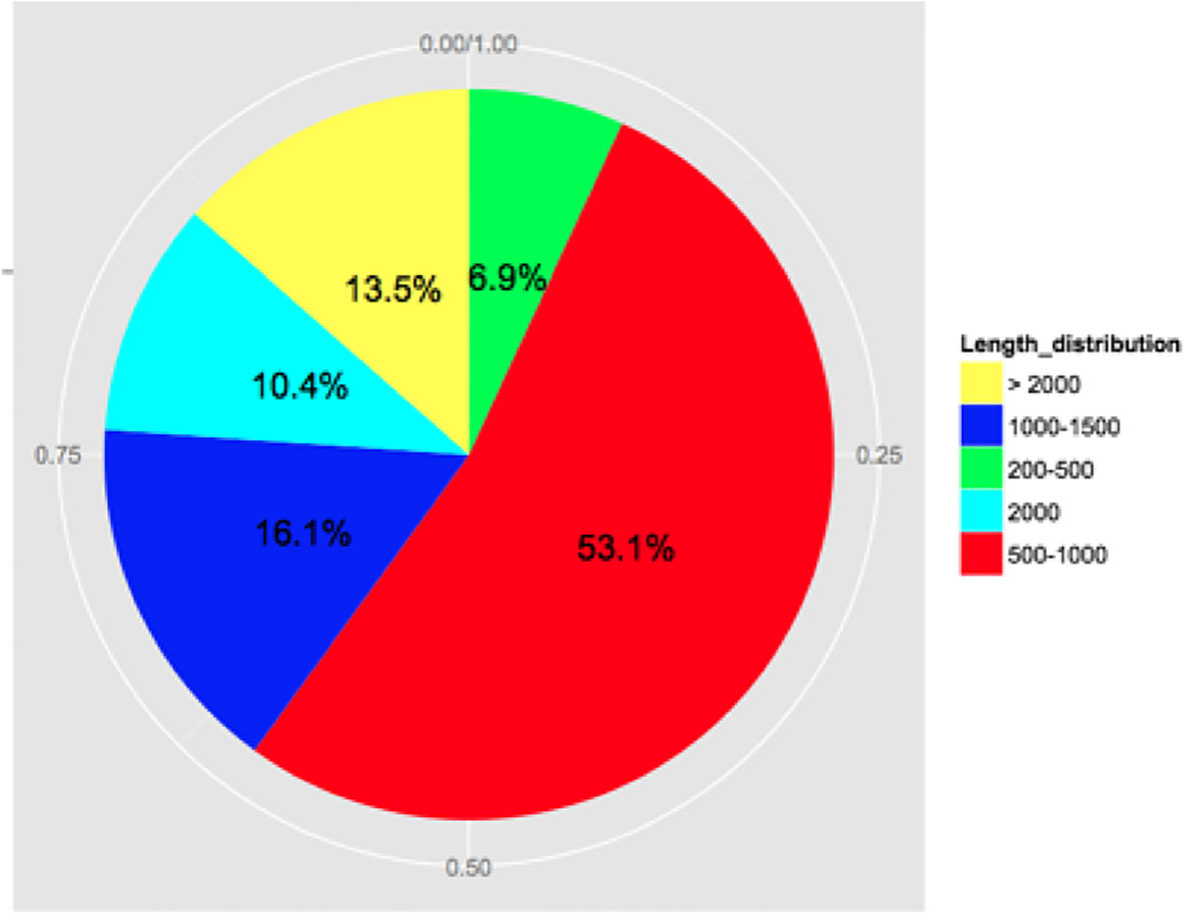
Length distribution of unigenes obtained from ‘HX’ radish fleshy root transcriptome

In this study, the cDNA library was constructed for HX-1, HX-2, and HX-3 genotypes (HX_RR_Root, HX_RW_Root, and HX_RP_Root, respectively). These three genotypes exhibit differential pigment contents in the taproot. The cDNA was prepared from the RNA samples isolated from the fleshy roots of radish exhibiting different phenotypes (RW, RP, and RR). The cDNAs were subjected to pair-end read (PE) sequencing using the HiSeq™ 2500 platform at the Beijing Genomics Institute (BGI, Shenzhen, China). In total, 66,356,258, 65,767,538, and 66,536,257 raw reads were obtained from the HX_RW_Root, HX_RP_Root, and HX_RR_Root libraries, respectively. After removal of low quality regions, adaptors and all possible contaminations, 63,371,122, 63,863,598, and 64,433,360 clean reads were generated, with a Q20 (base quality more than 20) percentage of almost 97.79, 96.86 and 97.74% in the HX_RW_Root, HX_RP_Root and HX_RR_Root libraries, respectively (Table 1). Subsequently, the clean reads were assembled into 198,342 contigs with an average length of 411 bp, which were assembled de novo into 34,927 unigenes (N50 = 1621 bp) with an average length of 768 bp (Fig. 1). The predominant length of the assembled unigenes ranged from 500 to 1500 bp (more than 69.2% of all unigenes) (Fig. 1). Moreover, 30,127 unigenes (86.26% of all unigenes) were annotated and matched to one or more of the public protein databases (Table 2).

**Table 1:** Statistics of output sequencing of radish ‘HX-1’ and advance inbred lines ‘HX-2’, ‘HX-3’ fleshy root transcriptome

Samples	HX_RW_root(HX-2)	HX_RP_root(HX-3)	HX_RR_root(HX-1)
Total raw reads	66, 356, 258	65,767,538	66,536,257
Total clean reads	63, 371, 122	63,863,598	64,433,360
Total clean nucleotides (nt)	5, 703, 400, 980	5, 747, 723, 820	5,799,002,400
Q20 percentage	97.79%	96.86%	97.74%
N percentage	0.00%	0.00%	0.00%
GC percentage	47.32%	46.97%	47.25%

Note: a Q20 percentage (percentage of sequences with sequencing error rate lower than 1%)

**Table 2:** Summary statistics of functional annotation of radish HX fleshy root unigenes in public databases

Public protein database	No. of unigene hit	Percentage (%)
NR	28, 758	82.34%
SwissProt	19, 036	54.50%
KEGG	18, 264	52.29%
COG	19, 888	56.94%
GO	26, 723	76.51%
ALL	30, 127	86.26%

**Fig. 2:**
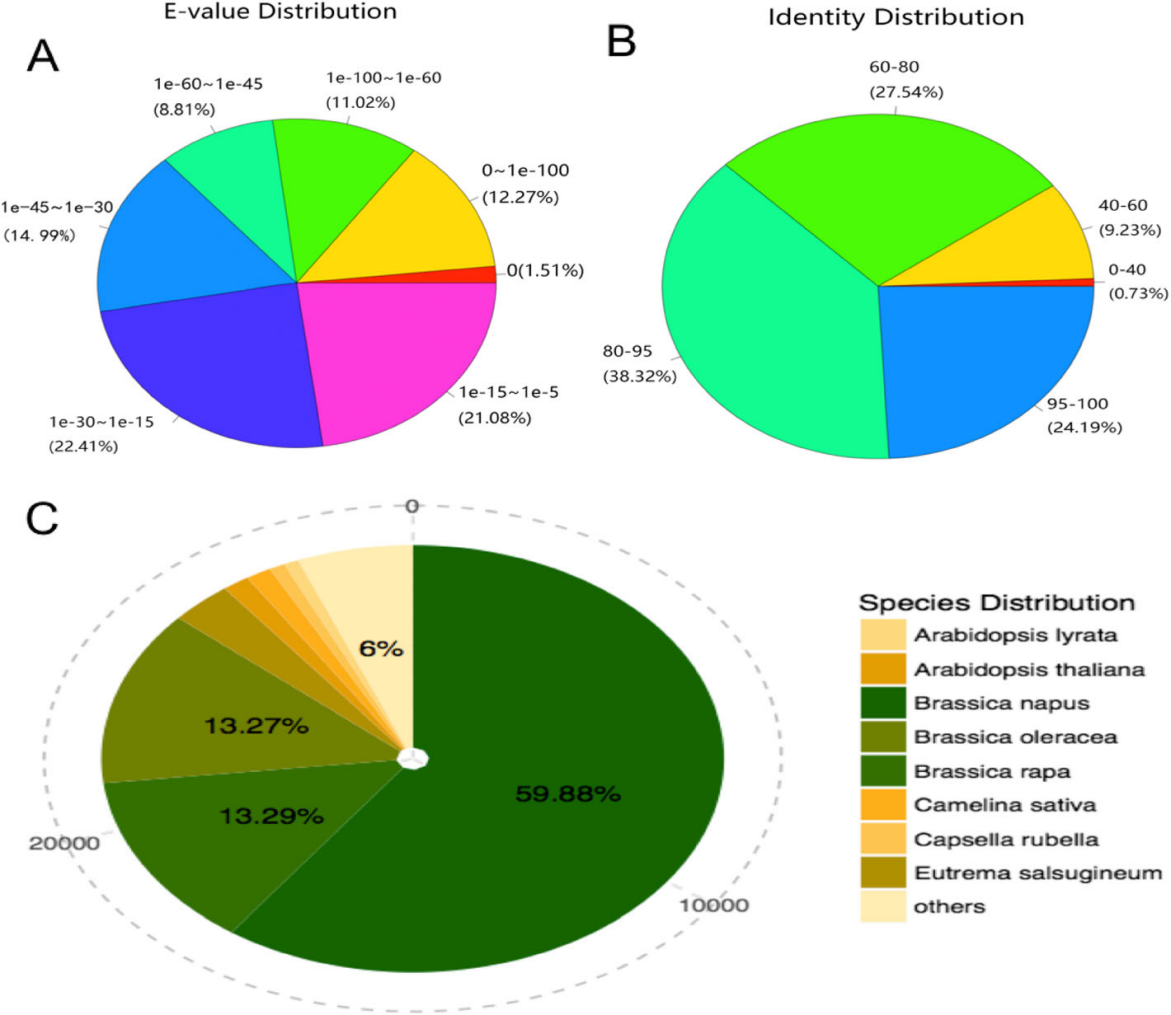
Characteristics of sequence homology of unigenes obtained from radish fleshy root Blasted against NCBI non-redundant (NR) database **a** E-value distribution of BLAST hits for matched unigene sequences, using an e-value cutoff of 1.0 e^− 5^. **b** Similarity distribution of top BLAST hits for each unigene. **c** Species distribution of the top BLAST hits.

The assembled sequences were subjected to BLASTx analysis. The analysis revealed that 67.90 and 66.39% of the clean reads exhibited strong (e-value < 1.0 e^− 45^) and moderate homology (e-value between 1.0 e^− 5^ and 1.0 e^− 45^) hits, respectively (Fig. 2a). Of those, 62.51% of the sequences with higher than 80% similarity were identified for the identity distribution pattern. Additionally, 65.86% of the sequences exhibited similarity between 60 and 95% (Fig. 2b). Most of the sequences exhibited 59.88, 13.29, 13.27, 6, and 3.45% similarity with the proteins of *Brassica napus*, *B. rapa*, *B. oleracea*, *Arabidopsis lyrata subsp. lyrata* and *A. thaliana,* respectively (Fig. 2c).

**Fig. 3:**
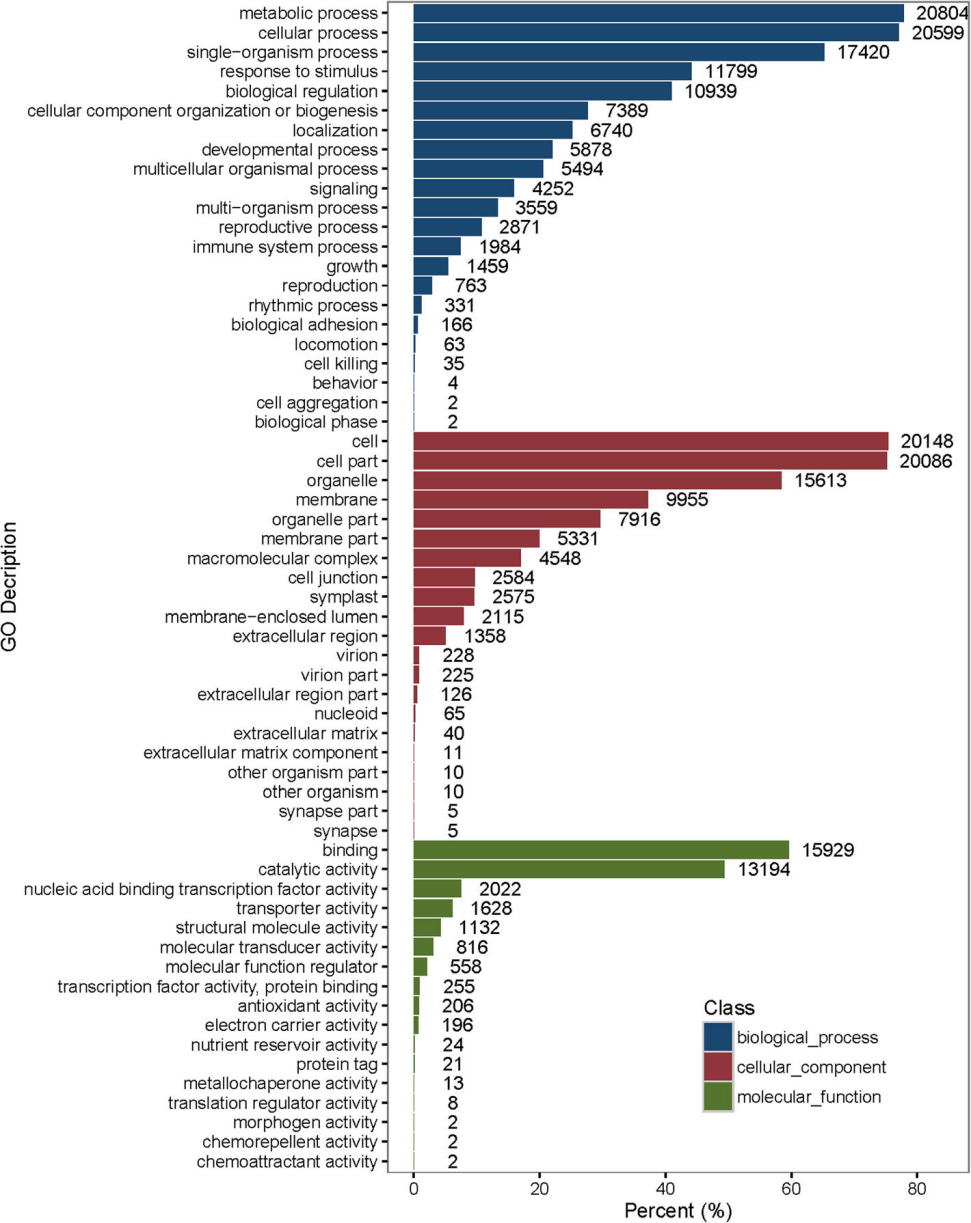
Gene ontology classification of the unigenes from radish ‘HX-1’ fleshy root transcriptome

In this study, the Gene Ontology (GO) terms were assigned to the assembled unigenes using the BLAST2GO program. The unigenes were categorized in three main GO categories: cellular component, molecular function, and biological process. In total, 26,723 unigenes (76.51%) were assigned at least one of the GO terms. Of those, the unigenes were predominantly assigned to the metabolic process (GO: 0008152, 20,804) and cellular process (GO: 0009987, 20,599). The unigenes categorized in the molecular function category were predominantly associated with binding (GO: 0005488, 15,929) and catalytic activity (GO: 0003824, 13,194). The unigenes categorized in the cellular components were associated with the organelles (GO: 0043226, 20,086) (Fig. 3). These findings demonstrated that the main GO classifications for the fundamental biological regulation and metabolism were identified from all the annotated unigenes.

**Fig. 4:**
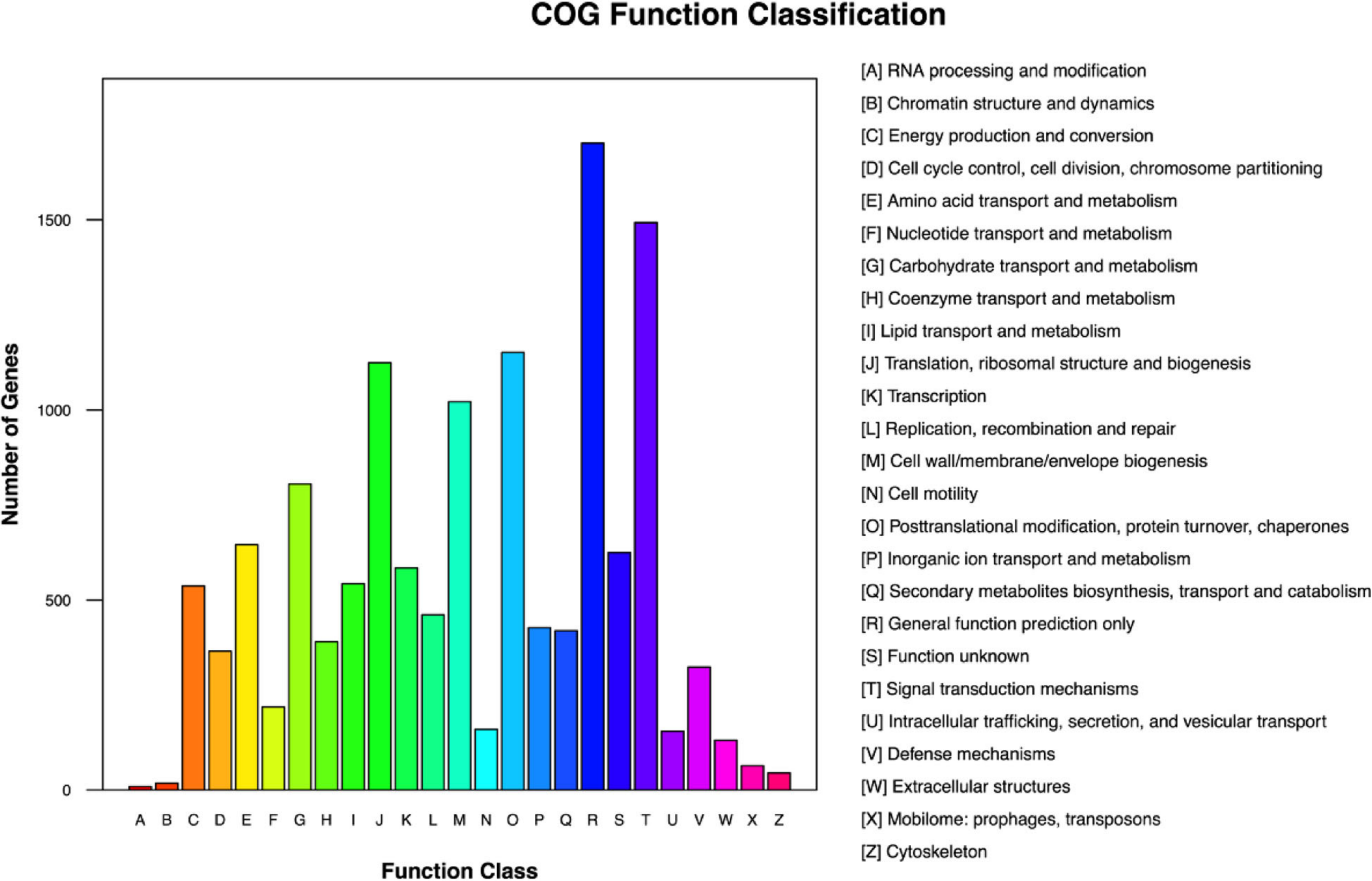
COG function classification of unigenes from radish ‘HX-1’ fleshy root transcriptome. Different colors represent the different categories of COG function classification (A-Z), and the details of those categories were listed in the right of graph

Of the 30,127 (56.94%) unigenes, 19,888 unigenes were categorized in 25 functional Clusters of Orthologous Groups (COG) clusters. Of those, the unigenes categorized in the cluster for “general functions prediction only”, which is related to the basic physiological and metabolic functions formed the largest group, whereas only few unigenes were categorized into the “RNA processing and modification” and “Chromatin structure and dynamics” clusters (Fig. 4). We proposed that most of the unigenes assigned to the clusters were associated with basic metabolism and biogenesis functions.

**Fig. 5:**
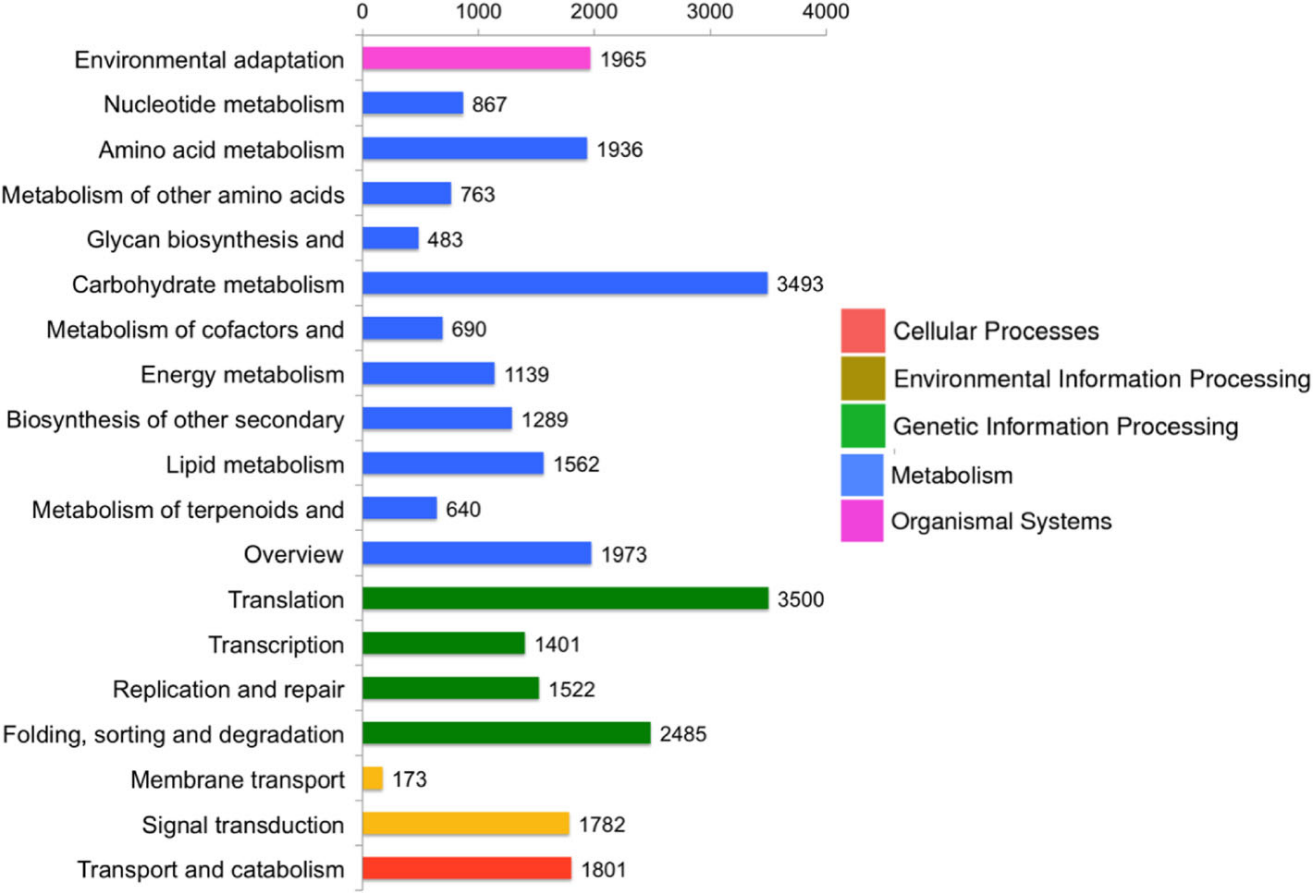
KEGG pathway annotation of assembled unigenes from radish ‘HX-1’ fleshy root transcriptome

The biological functions of genes involved in different networks can be systematically evaluated using the Kyoto Encyclopedia of Genes and Genomes (KEGG) pathway database. In this study, we mapped the assembled unigenes to the KEGG pathway database using the KEGG Orthology (KO) number. In total, 29,464 unigenes had significant matches to the database and were assigned to 138 KEGG pathways clustered in 5 main categories. The six dominant pathways were translation pathways (11.879%), folding, sorting and degradation pathway (8.434%), carbohydrate metabolic (11.855%), environment adaption (6.669%), signal transduction (6.048%), and transport and catabolism (6.113%) (Fig. 5).

**Fig. 6:**
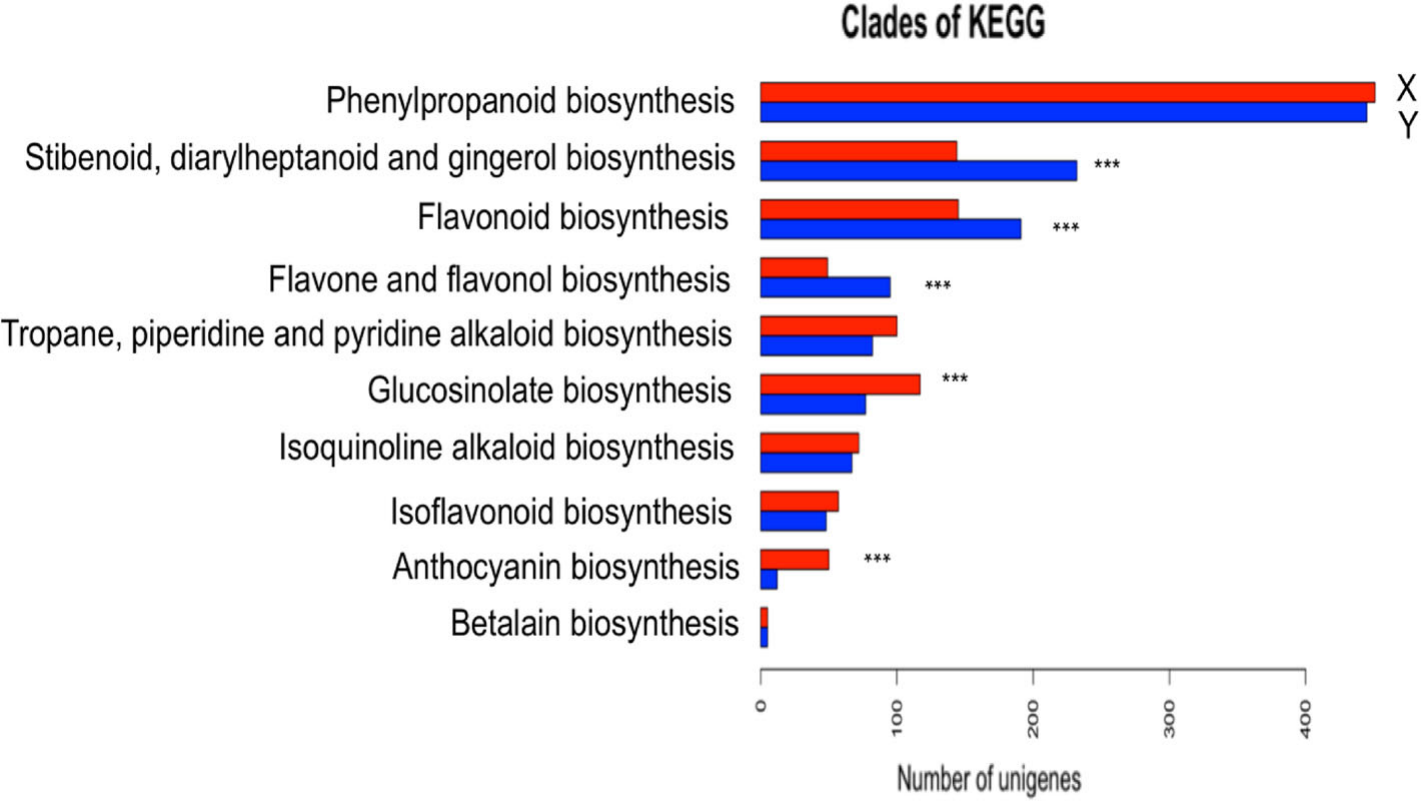
Comparative analysis of categories of secondary metabolite biosynthesis in ‘HX-1’ transcriptome with the results obtained from Liwang Liu. X and Y present categories of secondary metabolite biosynthesis in ‘HX-1’ transcriptome and radish transcriptome obtained from Liwang Liu et.al respectively

In the metabolism categories, expect for the carbohydrate metabolism, the biosynthesis of secondary metabolites was mainly grouped into 10 subcategories, especially for flavonoid biosynthesis, phenylpropanoid biosynthesis, glucosinolate biosynthesis, and betalain biosynthesis (Fig. 6). These genes associated with the secondary metabolite biosynthesis-related pathways (especially for anthocyanin biosynthesis and glucosinolate biosynthesis) may greatly enhance the potential utilization of HX radish taproot.

### Identification of candidate genes involved in the anthocyanin biosynthesis of radish

**Fig. 7:**
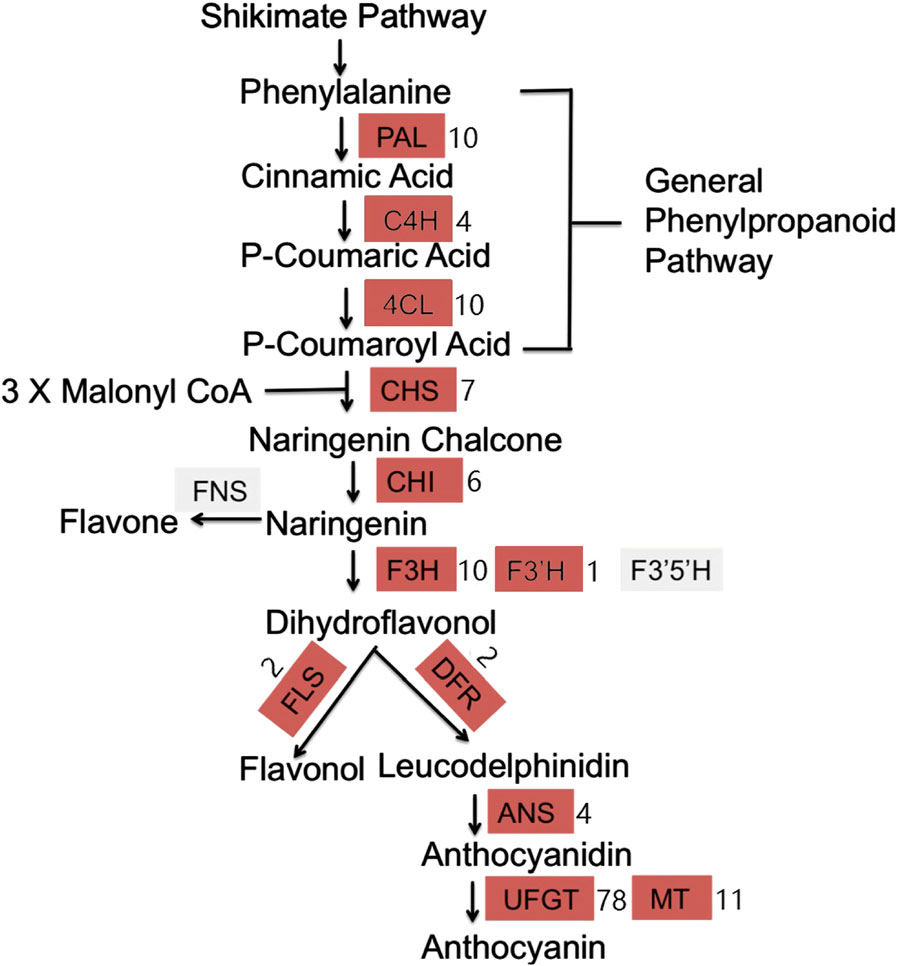
Assembled radish unigenes that may be involved in the anthocyanin biosynthesis pathway. The numbers following each gene name indicate the number of transcritome unigenes annotated to those genes. For transcript of *FNS* and *F3’5’H*, we used the gray color denoted that these transcripts could not detected in anthocyanin biosynthetic pathway in ‘Hongxin’ radish

Based on the KEGG database analysis, 29 ABRGs were identified, whose role in the anthocyanin biosynthetic pathway was examined (Fig. 7). In addition, we found that the number of unigenes (i.e., the final assembled sequence) often exceeded the number of expected transcripts (including isoforms). In this study, we listed the number of unigenes and genes for each transcript. The ABRGs included six *PAL* (10 unigenes), seven *4CL* syntenic genes (17 unigenes), four genes for *F3H* (10 unigenes), two genes each for *C4H* (2 unigenes) and *CHI* (6 unigenes), two genes for *CHS* (7 unigenes), *DFR* (2 unigenes) and *FLS* (2 unigenes), and one gene each for *ANS* (4 unigenes) and *F3’H* (1 unigene). Additionally, 176 unigenes related to methylation, glucosylation, and glycosylation were identified as follows: *UFGT*, *MT*, *GST* (Additional file 1: Table S1). However, the transcript of FNS and F3’5’H could not identify in anthocyanin biosynthetic pathway in ‘Hongxin’ radish.

### Validation and expression analysis of putative genes involved in anthocyanin biosynthesis

**Fig. 8:**
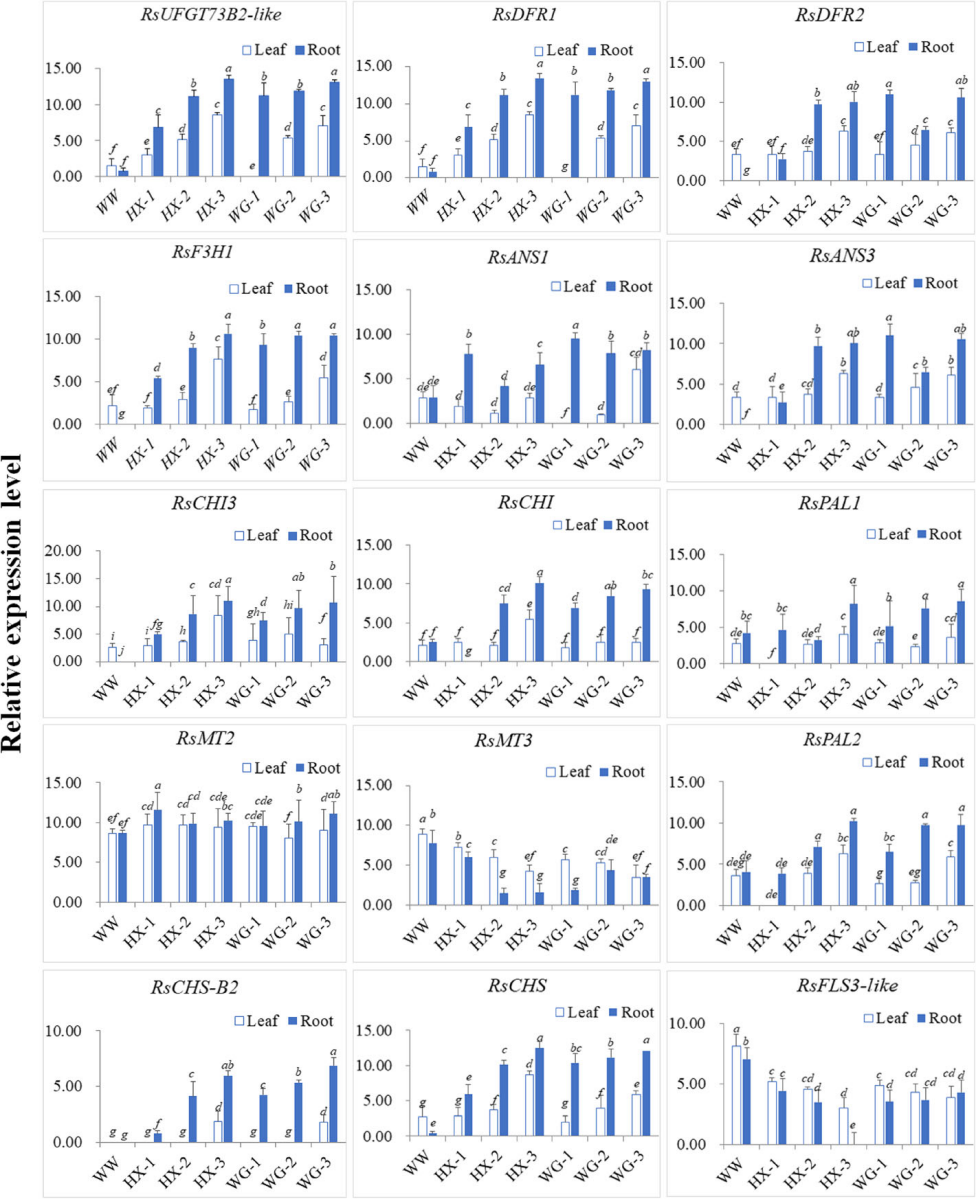
The quantitative real-time polymerase chain reaction (qRT-PCR) analysis of expression levels of 15 selected genes in different tissues of seven colour-variant radish genotypes. Three independent biological repeats were performed, and all data points were the means of three biological replicates ± standard error (SE). Variance analysis was carried out to evaluate differences between difference samples for root and leaf followed by multiple comparison using Duncan’s significant ranges least significant difference (SSR). Different letters indicate significance at *p* = 0.05 level. Of those, a, b, c, d, e, f, g means in a column with no superscript letter in common differ (*P* < 0.05), similar letters or common letters are not significantly different at the 5% level by Duncan’s multiple range test

The qRT-PCR analysis was performed to compare the dynamic expression patterns of 15 selected genes that were associated with anthocyanin biosynthesis. The leaves and young roots of seven colour-variant radish genotypes (WW, HX-1, HX-2, HX-3, WG-1, WG-2, and WG-3) were used for qRT-PCR analysis. Among these seven genotypes, WW genotype represents white radish with white skin and white flesh root. The HX-2 and HX-3 genotypes are advanced inbred lines of HX-1. The WG-2 and WG-3 genotypes are advanced inbred lines of WG-1. Of those, we observed that the expression of *RsPAL1* were similar in the leaves of all the colour-variant radishes, except for HX-1, but the expression level of *RsPAL1* was significantly upregulated in the roots of HX-3, WG-2, and WG-3; However, the expression levels of *RsPAL2* in the leaves and roots of HX-1, HX-2, HX-3, WG-1, WG-2, WG-2 were higher than those in the leaves and roots of WW. In addition, we found the expression levels of *RsCHS-B2*, *RsCHS*, *RsCHI*, *RsCHI3*, and *RsF3H1* were up-regulated in the root tissues of HX-3 and WG-3, but the expression of those genes was higher accumulated in the roots of HX-3 than WG-3. Additionally, the expressions of those genes were higher enriched in the leaves of HX-1, HX-2, and HX-3 than in the leaves of WW. However, we found the expression levels of *RsCHI* and *RsCHI3* were not significantly different between the leaves of WG-1, WG-2, and WG-3. The expression level of *RsCHS-B2* in the leaves of HX-3 was higher than that in the leaves of WG-3. The expression level of *RsFLS* in the leaves and roots of HX-1, HX-2, HX-3, WG-1, WG-2, and WG-3 was down-regulated when compared with that in the leaves and roots of WW. The *RsFLS* expression was especially decreased to minimal levels in the HX-3 and WG-3. More importantly, the expression levels of *RsDFR1* and *RsDFR2* were significantly up-regulated to varying degrees in the leaves and roots of HX-1, HX-2, HX-3, WG-1, WG-2, and WG-3, as well as the expression level of *RsANS3.*Of those, the expression levels of *RsDFR1* and *RsDFR2* were highest in the HX-3 and WG-3, respectively, but the expression level of *RsANS3* was only up-regulated in the leaves of WG-1, WG-2, and WG-3, with the highest expression level observed in WG-3, followed by WG-2 and WG-1. Additionally, the expression level of *RsANS1* in the leaves of HX-1, HX-2, HX-3, WG-1, WG-2, and WG-3 was up-regulated when compared with that in the leaves of WW. The expression level of *RsANS1* was the highest in the leaves of HX-3 and WG-3, whereas it was down-regulated in the roots of WG-1, WG-2, and WG-3. The highest expression of *RsANS1* was observed in the roots of WG-1. The expression level of *RsANS1* in the roots of HX-1, HX-2, and HX-3 was down-regulated. The expression level of *RsMT2* was not significantly different in the leaves and roots of HX-1, HX-2, HX-3, WG-1, WG-2, and WG-3 when compared with that in the leaves and roots of WW. However, the expression level of *RsMT3* was significantly downregulated in the leaves and roots of HX-1, HX-2, and HX-3. Among the HX-1, HX-2, and HX-3 genotypes, the expression level of *RsMT3* in the leaves was significantly down-regulated when compared to that in the roots. Nevertheless, the expression level of *RsUFGT73B2-like* was up-regulated in the HX-1, HX-2, HX-3, WG-1, WG-2, and WG-3 genotypes when compared with the expression level of *RsMT2* in WW. The expression pattern of *RsUFGT73B2-like* was similar among the coloured radish cultivars. Generally, the expression level of *RsUFGT73B2-like* was up-regulated among the coloured radish cultivars expressing high anthocyanin content, whereas the expression level of *RsMT3* was down-regulated (Fig. 8).

## Discussion

Anthocyanins are water-soluble pigments [1], which accumulate in many plant species. Anthocyanins are responsible for the red and purple colours in plants [2]. The ‘HongXin’ radish (*Raphanus sativus* L.), which contains natural red pigment (red radish pigment), is grown in Fuling district of Chongqing. However, the molecular mechanisms underlying anthocyanin synthesis for the formation of red pigment in the fleshy roots of HongXin radish are not well studied. In this study, the RNA was isolated from three radish genotypes exhibiting differential pigment contents. The cDNA was synthesized from the isolated RNA and subjected to pair-end read (PE) sequencing at the Beijing Genomics Institute (BGI, Shenzhen, China). Previous studies have demonstrated that PE sequencing can increase the depth of sequencing and the efficiency of de novo assembly [21, 22]. In this study, the number of clean reads was higher than that obtained from the cDNA libraries (prepared from 2 roots) reported in a previous study on radish transcriptome [23]. Moreover, the de novo transcriptome results were markedly more optimal than those reported in Wang et al. [24], where only the contigs with an average length of 299 bp and unigenes with an N50 length of 1095 bp were identified. Subsequently, the number of annotated unigenes in our study was higher than that in other non-model species reported in earlier studies (73.6% in blueberry, 58.01% in Chinese fir, and 58% in safflower flowers) [25, 26]. Of the annotated unigenes, the top BLAST hits were three species belonging to the Brassicaceae family (almost 86.44% of all assembled transcripts). This implied that the radish transcripts were assembled and annotated adequately in this study. In this study, the GO terms were assigned to the assembled unigenes using the BLAST2GO program and the unigenes were categorized into three main GO categories [27]. In total, 26,723 unigenes (76.51%) were assigned at least one of the GO terms. Importantly, we demonstrated that all the annotated unigenes were categorised into the main GO categories for fundamental biological regulation and metabolism. These results concurred with those reported in the tuberous root of sweet potato [28]and fleshy roots of radish (*Raphanus sativus* L.) [24].

The COG database provides information on the phylogenetic relationships of protein sequences from complete genomes of bacteria, algae, and eukaryotes [29]. In this study, the unigenes in the “general functions prediction only” cluster, which is related to basic physiological and metabolic functions, accounted for the largest group, whereas only few unigenes enriched in the “RNA processing and modification” and “Chromatin structure and dynamics” clusters. These results did not concur with those reported by Wang et al. [24], whereas the unigenes were mostly enriched in the “Post-translational modification, protein turnover, chaperones”, “Replication, recombination, and repair”, “Transcription”, and “Signal transduction mechanisms” clusters. We proposed that most of the unigenes were assigned to the clusters associated with the basic metabolism and biogenesis functions. In the metabolism categories, we observed that the assembled unigenes were more enriched in the anthocyanin and glucosinolate biosynthetic pathways when compared to the results obtained by Wang et al. [24]. However, the unigenes from the HX fleshy root transcriptome were less enriched in the flavonoid biosynthesis, flavone and flavonal biosynthesis, as well as stibenoid, diarylheptanoid and gingerol biosynthesis.

Anthocyanins are synthesised from phenylalanine by a series of enzymes in the phenylpropanoid pathway [9]. Of those, the genes encoding phenylalanine ammonia-lyase (*PAL*), cinnamic acid 4-hydroxylase (*C4H*), and 4-coumarate-CoA ligase (*4CL*) are involved in the phenylpropanoid pathway.Phenylalanine ammonia-lyase (PAL) enzyme, as the first committed step in the phenylpropanoid pathway that involved in the biosynthesis of polyphenolcompounds, such as flavonoids [30]. In this study, two genes (*RsPAL1* and *RsPAL2*) belonging to the PAL family were identified and validated by qRT-PCR. A previous study demonstrated that the *CHS*, *CHI*, *F3H*, and *F3’H* genes are involved in the flavonoid pathway and in the transfer of 4-coumaroyl CoA to dihydroflavonol [10]. In this study, the expression levels of *RsCHS-B2*, *RsCHS*, *RsCHI*, *RsCHI3*, and *RsF3H1* were found upregulated in the root and leaves of HX-3 and WG-3 (Fig. 8). However,*CHS* and *F3H* were found to be down-regulated in bicolor dahlia ‘Yuino’ [31] and colored yam [32] respectively. In grape, genes encoding *F3′5′H* and *F3′H* are especially expressed in skin of ripening red berries [33]. However, the transcript of *F3′5′H* could not detect in our study. During the synthesis of dihydroflavanols, the *RsDFR* and *RsANS* genes play critical roles in anthocyanin biosynthesis. Additionally, *RsFLS* regulates the conversion of dihydroflavanols to flavol. We found the expression level of *RsFLS* were downregulated in the root and leaves of HX-3 and WG-3. More importantly, the expression levels of *RsDFR1* and *RsDFR2* were significantly upregulated to varying degrees in the leaves and roots and highly enriched in the HX-3 and WG-3, respectively, as well as the expression level of the transcripts of *RsANS3 and RsANS1.* Previous study showed that over expression of *F3’H*, *DFR*, and *PAP1* were found involved in anthocyanin biosynthesis in treated Col-0 plants. However, the expression of *FLS1* was downregulated in co-cultivated plants [34]. These results were identical with the identified transcript of *F3’H*, *DFR and FLS* in our study. The *RsMT* and *RsUFGT* genes are two important regulators for glucosylation and methylation of anthocyanidins. These processes are important for the formation of stable anthocyanidins. However, the expression level of *RsMT2* was not significantly different in the leaves and roots in this study and the expression level of *RsMT3* was significantly downregulated in the leaves and roots. Nevertheless, the expression level of *RsUFGT73B2-like* was upregulated (Fig. 8). Previous studies have also demonstrated that the expression of *UFGT* is critical for fruit coloration in many plants, such as grape, strawberry and lychee [10, 14, 35, 36]. We proposed that *RsUFGT73B2-like* might be important regulator for glucosylation and methylation of anthocyanidins to formation of stable anthocyanidinsin HX-3 and WG-3.

## Conclusion

In this study, the assembled radish transcript sequences were analysed to identify the key ABRGs involved in the regulation of anthocyanin biosynthesis. Additionally, the expression patterns of candidate ABRGs involved in the anthocyanin biosynthesis pathway were validated by qRT-PCR. The results showed that the higher expression levels of *PAL*, *CHS, DFR* and *UFGT* in ‘HX’ and ‘WG’ leaf/root than in those of ‘WW’, suggested that these genes are responsible for color formation in the leaf and root of ‘HX’ and ‘WG’ radish. This study will improve our understanding of the molecular mechanisms involved in the biosynthesis and metabolism of anthocyanin in radish.

## Methods

### Plant materials

In this study, we used one inbred line (HX-1), and two advanced inbred lines (HX-2 and HX-3) of radish (*Raphanus sativus* L.). The HX-1, HX-2, and HX-3 lines were inbred through self-pollination for more than 6 generations. The materials were planted under normal conditions at the Yihe Breeding Station of Yangtze Normal University, Chongqing City, China and placed in our lab. The HX-1, HX-2, and HX-3 genotypes exhibit red skin and red fleshy root (RR), red skin and white fleshy root (RW) and red skin and pinkly fleshy root (RP) phenotypes, respectively (Additional file 2: Fig. S1). The seedlings were cultured in a growth chamber under the following growth conditions: 14 h light at 25 °C and 10 h darkness at 18 °C. For Solexa analysis, the taproots of the HX-1, HX-2, and HX-3 genotypes were sampled at mature stages. The skin and flesh were simultaneously collected. The samples were cut into small cubes at mature stage prior to experiments. All samples were washed with distilled water, frozen in liquid nitrogen, and stored at − 80 °C for RNA extraction.

### RNA extraction and Illumina sequencing

Total RNA was extracted from the taproot samples using the RNAprep pure Plant Kit (Tiangen Biotech Co., Ltd., China). The RNA samples were treated with RNase-free DNase I to degrade the DNA (Takara, Japan). The three cDNA libraries (HX_RW_Root, HX_RP_Root, and HX_RR_Root) were constructed by Beijing Genomics Institute (BGI, Shenzhen, China). The clean reads were obtained from the raw reads generated by Illumina Hiseq™ 2500 and de novo assembled using the Trinity progra m[37].

### Functional annotation and classification of the assembled transcripts

In this study, we used five public protein databases: NCBI non-redundant protein (Nr), Swiss-Prot protein, clusters of orthologous groups (COG), gene ontology (GO), and Kyoto Encyclopedia of Genes and Genomes (KEGG) databases. The assembled transcripts were compared with the databases using BLASTx analysis with a cut-off e value of 10^− 5^. When the results were conflicting between the databases, the following priority order was used: Nr, Swiss-Prot, KEGG, GO and COG. For the Nr annotations, we used the BLAST2GO program to assign the unique assembled transcripts to the GO categories (comprised of biological processes, molecular functions, and cellular components )[38]. Subsequently, functional classification was performed using the WEGO softwar e[39].In brief, GO functional classification of all sequences were performed to view the distribution of gene functions of the species at the macro level. Subsequently, the number of sequences associated with every term were calculated through mapped all of the annotated sequences to GO terms.

### qRT-PCR analysis

The young leaves and roots of seven colour-variant radish genotypes (‘WW’, ‘HX-1’, ‘HX-2’, ‘HX-3’, ‘WG-1’, ‘WG-2’ and ‘WG-3’) were used to identify the putative genes associated with anthocyanin biosynthesis. The WW genotype represents white radish with white skin and white flesh root. HX-2 and HX-3 represent advanced inbred lines of HX-1 inbred line; WG-2 and WG-3 represent advanced inbred lines of WG-1 inbred line (Additional file 2: Fig. S1). The qRT-PCR analysis was performed using the SYBR premix Ex Taq kit (TaKaRa, China) on ABI 7500 Real-Time System (Applied Biosystems) platform. The primers are designed by Primer 5.0 software for qRT-PCR experiments and radish gene (Actin) is used as a standard control (Additional file 1: **Table S2**). The amplification programs were performed according to the standard protocol of the ABI7500 system, and conducted in triplicate as mentioned by Jian et al. [40]. The relative quantitative method (2^-△△CT^) was used to calculate the fold change in the expression levels of target genes [41].

### Statistical analysis

Variance analysis was carried out to evaluate differences between difference samples for root and leaf followed by multiple comparison using Duncan’s significant rangesleast significant difference (SSR), which was performed with SPSS statistical software to detect significant differences among the relative expression levels of the genes. Different letters indicate significance at *p* = 0.05 level. Of those, a, b, c, d, e, f, g means in a column with no superscript letter in common differ (*P* < 0.05), similar letters or common letters are not significantly different at the 5% level by Duncan’s multiple range test.

## Supplementary information

**Additional file 1: Table S1.** Identification of pupative genes involved in anthocyanin biosythesis in ‘Hongxin’ radish. **Table S2.** List of primers for qRT-PCR analysis of anthocyanin synthesis-related genes (ASRGs) identified in *Raphanus sativus*.

**Additional file 2: Figure S1.** Fleshy roots from seven types of radish are shown in A-G, including WW (white radish white skin and white fleshy root), HX-1 (Hongxin red skin and white fleshy root), HX-2 (Hongxin red skin and pink fleshy root), HX-3 (Hongxin red skin and red fleshy root), WG-1 (Waguan red skin and white fleshy root), WG-2 (Waguan red skin and pinkly fleshy root) and WG-3 (Waguan red skin and red fleshy root).

## Data Availability

The datasets supporting the conclusions and description of a complete protocol are included within the article.

## Abbreviation

4CL: 4-coumarate: CoA ligase
ANS: Anthocyanin synthase
ASRGs: Anthocyanin synthesis-related genes
BP: Biological process
C4H: Cinnamate 4-hydroxylase
CC: Cellular component
CHI: Chalcone isomerase
CHS: Chalcone synthase
DEGs: Differential expression genes
DFR: Dihydroflavonols 4-reductase
F3’5H: Flavonoid 3,5 –hydroxylase
F3H: Flavanone 3-hydroxylase
FLS: Flavcnol synthase
FNS: Flavone synthase
FPKM: Fragments per kilobase of transcript per million mapped reads
GO: Gene ontology
KEGG: Kyoto encyclopedia of genes and genomes
MF: Molecular function
MT: Metallothionein-like protein
NCBI: National center for biotechnology information
PAL: Phenylalanine ammonia-layse
RP: Red skin and pinky flesh root
RR: Red skin and red flesh root
RW: Red skin and white flesh root
UFGT: UDP-glucose: Flavonoid 3-glucosyltransferase
WW: White skin and white flesh
