# Supplementary material for: De novo transcriptome sequencing of radish (Raphanus sativus L.) fleshy roots: analysis of major genes involved in the anthocyanin synthesis pathway
Source: BMC Mol Cell Biol. 2019 Oct 23;20:45. doi: 10.1186/s12860-019-0228-x (PMC6813128; doi:10.1186/s12860-019-0228-x)
Supplement: Supplementary file 2 — Additional file 2: Figure S1. Fleshy roots from seven types of radish are shown in A-G, including WW (white radish white skin and white fleshy root), HX-1 (Hongxin red skin and white fleshy root), HX-2 (Hongxin red skin and pink fleshy root), HX-3 (Hongxin red skin and red fleshy root), WG-1 (Waguan red skin and white fleshy root), WG-2 (Waguan red skin and pinkly fleshy root) and WG-3 (Waguan red skin and red fleshy root). [file 12860_2019_228_MOESM2_ESM.docx]

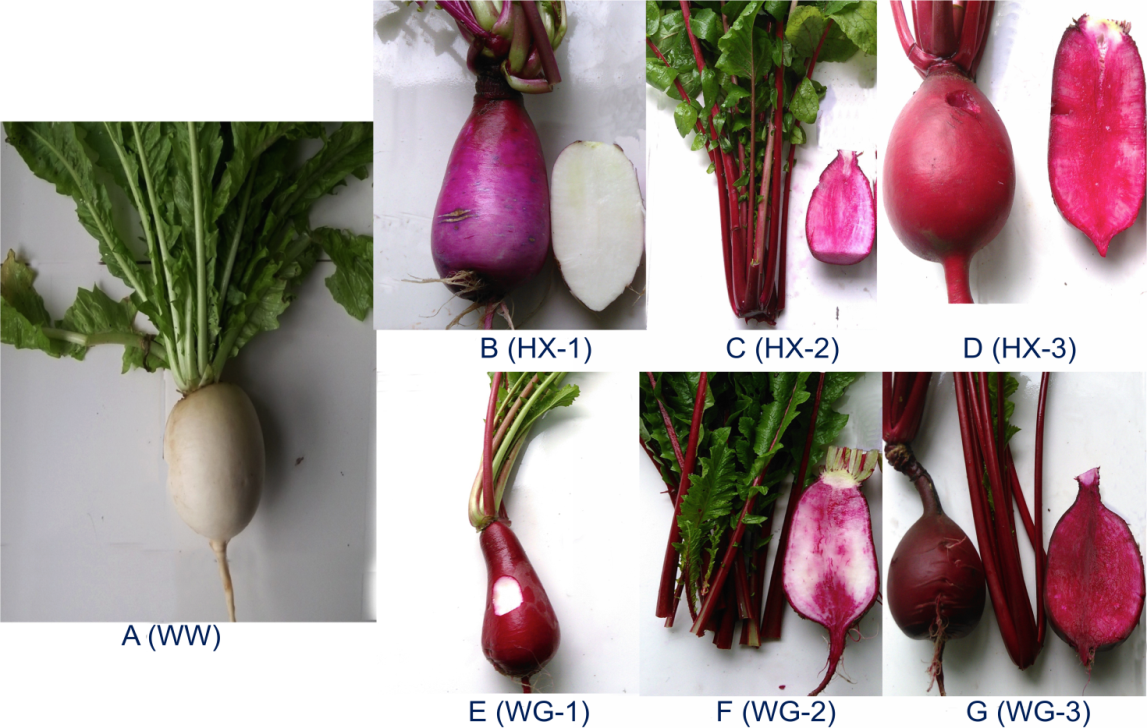


**Fig. S1** Fleshy roots from seven types of radish are shown in A-G, including WW (white radish white skin and white fleshy root), HX-1 (Hongxin red skin and white fleshy root), HX-2 (Hongxin red skin and pink fleshy root), HX-3 (Hongxin red skin and red fleshy root), WG-1 (Waguan red skin and white fleshy root), WG-2 (Waguan red skin and pinkly fleshy root) and WG-3 (Waguan red skin and red fleshy root)
